# Association of professional identity, job satisfaction and burnout with turnover intention among general practitioners in China: evidence from a national survey

**DOI:** 10.1186/s12913-021-06322-6

**Published:** 2021-04-26

**Authors:** Tao Zhang, Jing Feng, Heng Jiang, Xin Shen, Bo Pu, Yong Gan

**Affiliations:** 1 Department of Health Management, School of Medicine and Health Management, Tongji Medical College, Huazhong University of Science and Technology, Wuhan, Hubei China; 2 Department of Social Medicine and Health Service Management, School of Public Health, Hangzhou Normal University, Hangzhou, Zhejiang, China; 3 Department of Social Medicine and Health Management, School of Public Health, Tongji Medical College, Huazhong University of Science and Technology, No. 13 Hangkong Road, Wuhan, 430030 Hubei China; 4 Centre for Alcohol Policy Research, School of Psychology and Public Health, La Trobe University, Melbourne, Victoria Australia; 5 Centre for Health Equity, Melbourne School of Population and Global Health, University of Melbourne, Melbourne, Victoria Australia; 6 School of Business and Tourism, Sichuan Agricultural University, Chengdu, Sichuan China

**Keywords:** Primary care, General practitioner, Turnover intention, Job satisfaction, Professional identity, Burnout

## Abstract

**Background:**

The complex interrelationships between professional identity, job satisfaction, burnout, and turnover intention among general practitioners (GPs) are insufficiently understood in China. This study aimed to investigate the interrelationships between professional identity, job satisfaction, burnout, and turnover intention in China, and to examine whether job satisfaction and burnout played mediating roles between professional identity and turnover intention.

**Methods:**

A cross-sectional survey was conducted between October, 2017 and February, 2018 in China. The participants were selected using a multistage stratified random sampling method. Data were collected with a self-administered questionnaire from 3236 GPs (response rate, 99.8%) working in community health institutions in China. Professional identity was measured by the 13 items scale, and job satisfaction scale with an 11-item designed by Shi et al. was employed. Burnout was measured using a 22-item Maslach Burnout Inventory-Human Services Survey, and turnover intention was measured with a 6 items scale. Descriptive statistics were calculated and groups’ differences were estimated Student’s *t*-test and analyses of variance. Pearson’s correlation analysis was used to assess the degree of correlation among different dimensions of professional identity, job satisfaction, burnout, and turnover intention. Structural equation modeling analysis was applied to examine the interrelationships among these study variables based on the hypothesized model.

**Results:**

The proposed model achieved a good model fit. Job satisfaction had a direct negative effect on turnover intention (*β* = − 0.38, *P* < 0.001), burnout had a direct positive effect on turnover intention (*β* = 0.37, *P* < 0.001), and professional identity had an indirect negative effect on turnover intention through the mediating effect of job satisfaction and burnout.

**Conclusions:**

Our study elucidated the pathways linking professional identity, job satisfaction, and burnout to turnover intention of GPs. This revealed that turnover intention was significantly affected by job satisfaction and burnout, and the effects of professional identity on turnover intention can be mediated by job satisfaction and burnout.

**Supplementary Information:**

The online version contains supplementary material available at 10.1186/s12913-021-06322-6.

## Background

Providing adequate and high-quality primary healthcare has been considered as a priority in the health care system with rapid population aging around the world. General practitioners (GPs), gatekeepers for residents’ health, are the main providers of primary healthcare services and supposed to provide a wide range of economical, convenient and highly comprehensive health services [1, 2]. These basic health services can sustain the equality and efficiency of overall health care services, as well as control the increase in medical costs [3]. Therefore, many developed countries have established a relatively comprehensive GPs system and paid more attention to GPs’ career development and training.

Since China’s new round of health system reform in 2009, improving the capability of primary healthcare institutions has been positioned as the core task of the reform [4]. Thereafter, Chinese government launched a new plan for the GP system with a goal of providing five GPs for every 10,000 people by 2030, which aimed to shift patients away from overcrowded hospitals and take the strain off the current healthcare system [5]. In order to achieve such goal, many efforts have been taken to train and recruit GPs. For example, the new “5 + 3” model of medical education was introduced in 2015. Medical students who have completed a 5-year bachelor’s degree can choose to finish a 3-year standardized clinical training if they intend to become GPs [6, 7]. Not surprisingly, these efforts have created an encouraging progress. For instance, the number of GPs per 10,000 population increased from 0.8 in 2012 to 2.2 in 2018 [8]. However, the shortage of health workers and high turnover rate among on-post GPs still are the urgent issue in primary healthcare sectors in China [9].

Previous studies showed that GPs in primary health institutions [community health centres (CHCs) and township health centres (THCs)] had a high-level turnover rate, especially in poor regions [10, 11]. Although Chinese government has attempted to improve working conditions and levels of wage for primary health workers, GPs’ turnover intention still remained at a high level [12]. Thus, understanding factors associated with GPs’ turnover intention become particularly important in current Chinese context.

Several studies have investigated potential factors correlated to GPs’ turnover intention, such as low income, high work stress, high professional title and limited professional development opportunities [1, 11, 13–18]. However, these studies mainly focused on demographics factors and job characteristics, and many other potential factors (such as professional identity, job satisfaction and burnout) were neglected. The heuristic model of the employee withdrawal decision process proposed by Mobley indicated a significant correlation between job satisfaction and turnover intention [19]. Simultaneously, studies of the attitude theory in psychology revealed that employees’ perception and evaluation on their works (job satisfaction) have an negative association with their emotional response (burnout) [20, 21]. Additionally, professional identity is other important factor which is referred to one’s perception on social significance and value of works he/she engages, and has been demonstrated an evident association with job satisfaction, burnout and turnover intention among nurses and specialists [22, 23]. Therefore, job satisfaction, burnout, and professional identity should be considered as important factors for turnover intention among GPs. Also, it is assumed that associations between these variables and turnover intention are not only direct, but some indirect mediating effects could exist. For example, the relationship between job satisfaction and turnover intention may be mediated by burnout. However, no studies provide a clear insight on these complex interrelationships among Chinese GPs working in the community health institutions (CHIs) based on a national representative sample.

**Fig. 1:**
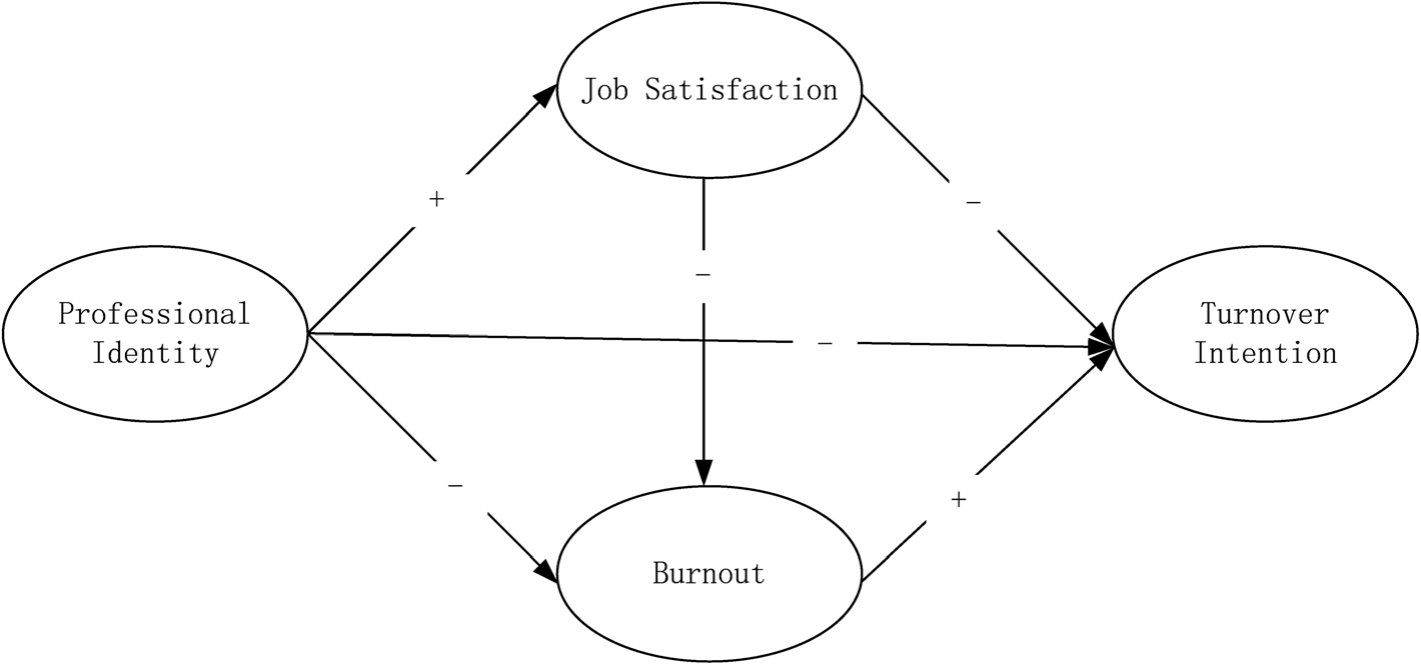
Hypothetical model of relationships between professional identity, job satisfaction, burnout, and turnover intention among Chinese urban GPs

Based on above review, this study aimed to fill the research gap by analysing the interrelationships between professional identity, job satisfaction, burnout, and turnover intention among Chinese GPs using a national survey data. As illustrated in Fig. 1, we proposed the following hypotheses: (1) professional identity and job satisfaction have a negative association with GP’s turnover intention, while the correlation between burnout and turnover intention is positive; (2) the relationship between professional identity and turnover intention would be mediated by both job satisfaction and burnout; (3) burnout also creates a mediation effect on the relationship between job satisfaction and turnover intention.

## Methods

### Study population and sampling

A cross-sectional survey was conducted in China from 10th October 2017 to 28th February 2018. In this survey, a multistage sampling strategy was applied for data collection. Firstly, we chose 4 provinces and municipalities from each of three different levels of economic development and geographical regions, namely: Shanghai, Beijing, Guangdong, and Zhejiang in the eastern region; Hubei, Anhui, Heilongjiang, and Henan in the central region; Sichuan, Chongqing, Guizhou, and Yunnan in western China region. Secondly, 30 CHIs including CHCs and community health stations (CHSs) were randomly selected according to agency code from each province, which resulted in a total of 360 participating CHIs. Thirdly, according to the number of GPs and scales of CHIs, 40% of the on-post GPs with at least one-year work experience from each sampled CHIs were randomly invited to participate in the survey. In order to capture a representative sample, we approached potential participants who were on duty during the survey period. In total, 3244 urban GPs were invited to complete a self-administered questionnaire (Additional file 1) [24]. Of those invited participants, 3236 (99.75%) returned valid questionnaires for data analyses. The excluded questionnaires (*n* = 8) contained incomplete data on the four scales measurement. The remaining sample was large enough to establish our model containing 15 variables [25]. A power analysis for the not close model fit (α = 0.05; statistical power = 0.95; null root mean square error of approximation (RMSEA) = 0.05; and alternative RMSEA = 0.08 [26]; df = 39) showed the minimum sample size of 402 [27]. Thus, the sample size was sufficient in our study.

The study protocol was approved by the Ethics Committee of the Tongji Medical College Institutional Review Board, Huazhong University of Science and Technology, Wuhan, China (No. [2018] IEC (S186)). All the participating doctors gave their informed consent before completing the questionnaire.

### Instrument and measurement

#### Demographic characteristics

Demographic information captured in the survey included gender, age, marital status, education, income level, professional title, employment status, work tenure and working hours (Table 1). Self-rated income level was measured by an item on “what was the level of your income in your local city or region?” (below the moderate, at the moderate level, or higher than the moderate). Work hours referred to average working hours per day for GPs.

**Table 1:** Characteristics of respondents and differences in professional identity, job satisfaction, burnout and turnover intention scores among different characteristics groups (mean ± SD)

Characteristics of respondents	***N (%)***	Professional identity	Job satisfaction	Burnout	Turnover intention
**Gender**					
Male	1170 (36.2)	51.46 ± 6.91	36.47 ± 7.75	61.10 ± 22.45	15.42 ± 4.49
Female	2066 (63.8)	51.09 ± 7.13	37.67 ± 6.85	59..56 ± 20.60	14.86 ± 4.22
*t*		1.43	−4.56	1.98	3.53
*p*		0.15	< 0.001	0.04	< 0.001
**Age (years)**					
≤ 30	689 (21.3)	50.51 ± 7.05	37.57 ± 7.58	63.64 ± 20.79	15.33 ± 4.31
31–40	1469 (45.4)	51.07 ± 7.12	37.01 ± 7.17	61.62 ± 20.96	15.57 ± 4.01
41–50	914 (28.2)	51.69 ± 7.11	37.01 ± 7.07	56.86 ± 21.44	14.45 ± 4.52
≥ 51	164 (5.1)	52.89 ± 5.58	39.17 ± 6.40	49.96 ± 20.29	12.76 ± 4.90
*F*		6.97	5.19	29.05	29.94
*p*		< 0.001	0.001	< 0.001	< 0.001
**Marital status**					
Non-married	382 (11.8)	50.07 ± 7.28	37.33 ± 7.79	64.46 ± 21.68	15.43 ± 4.31
Married	2771 (85.6)	51.35 ± 7.00	37.21 ± 7.12	59.57 ± 21.15	15.04 ± 4.31
Others	83 (2.6)	52.22 ± 7.37	37.85 ± 7.49	58.25 ± 22.36	14.04 ± 4.71
*F*		6.43	0.35	9.19	3.71
*p*		0.001	0.70	< 0.001	0.25
**Education level**					
Associate’s degree or below	918 (28.4)	51.15 ± 7.04	38.05 ± 7.39	57.71 ± 21.78	14.26 ± 4.65
Bachelor’s degree	2139 (66.1)	51.25 ± 6.98	36.92 ± 7.07	61.13 ± 21.06	15.32 ± 4.15
Master’s degree or above	179 (5.5)	51.22 ± 8.00	36.91 ± 7.61	60.32 ± 20.71	16.07 ± 4.02
*F*		0.07	8.06	8.32	24.73
*p*		0.93	< 0.001	< 0.001	< 0.001
**Income level**					
Low	2281 (70.5)	50.56 ± 7.22	36.13 ± 7.10	61.81 ± 21.49	15.54 ± 4.21
Middle	864 (26.7)	52.42 ± 6.28	39.86 ± 6.64	55.89 ± 20.42	13.99 ± 4.30
High	91 (2.8)	54.06 ± 7.74	40.09 ± 7.95	57.73 ± 18.98	13.34 ± 5.05
*F*		27.69	96.48	25.17	48.59
*p*		< 0.001	< 0.001	< 0.001	< 0.001
**Professional title**					
Junior	1419 (43.9)	50.87 ± 7.01	37.65 ± 7.56	60.88 ± 21.49	15.08 ± 4.41
Middle	1412 (43.6)	51.30 ± 7.11	36.68 ± 6.92	60.36 ± 21.22	15.17 ± 4.23
Senior	405 (12.5)	52.18 ± 6.96	37.71 ± 6.82	56.59 ± 20.60	14.61 ± 4.36
*F*		5.54	7.18	6.57	2.60
*p*		0.01	0.001	0.001	0.07
**Employment status**					
Permanent employee	2185 (67.5)	51.18 ± 7.13	37.03 ± 7.07	60.67 ± 21.01	14.86 ± 4.21
Contract employee	789 (24.4)	51.31 ± 6.88	37.84 ± 7.21	58.69 ± 21.78	15.44 ± 4.42
Others	262 (8.1)	51.27 ± 6.99	37.20 ± 8.23	59.78 ± 22.09	15.63 ± 4.82
*t*		0.10	3.63	2.54	7.65
*p*		0.90	0.26	0.79	< 0.001
**Work tenure (years)**					
1–3	993 (30.7)	50.45 ± 7.26	37.14 ± 7.53	61.48 ± 21.73	15.46 ± 4.35
4–10	1607 (49.7)	51.63 ± 6.78	37.38 ± 7.20	60.07 ± 20.66	15.08 ± 4.20
≥ 11	636 (19.7)	51.39 ± 7.32	37.03 ± 6.71	58.12 ± 22.06	14.39 ± 4.52
*F*		8.77	0.66	4.84	12.05
*p*		< 0.001	0.51	0.01	< 0.001
**Working hours (hours)**					
≤ 8	2374 (73.4)	51.16 ± 6.78	37.76 ± 7.08	58.80 ± 20.70	14.79 ± 4.32
≥ 9	862 (26.6)	51.40 ± 7.55	35.79 ± 7.37	63.73 ± 22.49	15.81 ± 4.26
*t*		0.78	47.84	34.18	35.39
*p*		0.37	< 0.001	< 0.001	< 0.001

#### Turnover intention

Turnover intention scale (TIS-6), developed by Michaels et al. and revised by Li et al. for the Chinese population, was used to measure GPs’ intention to resign in this study [28, 29]. This scale included six items, for example “Have you considered resigning from your current job?”. Items 1 and 6 measured the intent to resign from the current job (TIS-I); items 2 and 3 represented the motivation to search for other jobs (TIS-II); and items 4 and 5 indicated the probability of obtaining a new job (TIS-III). Each item was rated with a four-point Likert scale, ranging from 1 (never) to 4 (always). Consequently, the total score of six items ranged from 6 to 24 and a higher score indicated stronger turnover intention. The scores of 0–6, 7–12, 13–18, and 19–24 were assigned for the low, moderate, moderate to high and high degree of turnover intention, respectively [29]. In this study, the Cronbach’s alpha coefficient for the scale was 0.89, indicating a good internal consistency.

#### Burnout

Burnout was measured using the Chinese version of anonymous Maslach Burnout Inventory-Human Services Survey (MBI-HSS) questionnaire including three subscales: emotional exhaustion (EE, 9 items), depersonalization (DP, 5 items), and personal achievement (PA, 8 items) [30]. EE describes the feelings of being emotionally overextended and exhausted by one’s work; DP measures an unfeeling and impersonal response towards recipients of one’s care or service and PA indicates feelings of competence and successful achievement in one’s work with people. Each item in three subscales is scored according to how often the statement is experienced, from ‘never’ (0) to ‘every day’ (6). Higher scores on the EE and DP subscales indicate the higher degrees of burnout, while PA is inversely correlated with burnout. The reversed scores of the PA were used to estimate the burnout. The summary scores for burnout range from 0 to 132, with high scores representing high degrees of burnout. The Cronbach’s alphas for the MBI-HSS, EE, DP, and PA were 0.89, 0.94, 0.90 and 0.88, respectively.

#### Job satisfaction

Job satisfaction scale developed by Shi et al. was used to assess GPs’ satisfaction. A total of 11 items in this scale were rated using a five-point ordinal scale ranging from 1 (very dissatisfied) to 5 (very satisfied). These items can be divided into 3 dimensions: satisfaction on relationship (4 items), satisfaction on personal development (3 items) and satisfaction on basic demand (4 items) [31]. The total scores of job satisfaction ranged from 11 to 55, with higher scores indicating a higher level of satisfaction. The Cronbach’s alpha for this scale, material satisfaction, relationship satisfaction, and growth satisfaction was 0.91, 0.82, 0.77, and 0.86, respectively.

#### Professional identity

According to Zhao et al. and Wu’s study [32, 33], the Chinese version of professional identity scale in this study was measured using 13 items. Each statement was asked to response from very disagree (1) to very agree (5). This scale can be divided into 2 dimensions: professional value (9 items) and professional efficacy (4 items). Professional value tended to measure GPs’ perception on social value and meaning of their works, for example, “The work I do is important to the health of the patient”. Professional efficacy focused on GPs’ perception on personal accomplishment and honour in regard to their works, for example “Others praise my profession, just like praise to me”. The item scores were summed to provide a composite score of professional identity (range: 13 to 65) and a high score represented higher professional identity. In the present study, the Cronbach’s alpha for this scale was 0.91.

### Data collection and quality control

The questionnaire was designed based on a literature review, group discussions, and mock interviews. Furthermore, to improve the quality of the questionnaire, a pilot study was conducted in Wuhan’s CHIs. A web link of the online questionnaire, created by the Questionnaire Star software, was then disseminated to GPs through WeChat (the biggest communication platform in China with over one billion users, similar to WhatsApp in western countries). The data were entered into the web-based database by specialized investigators to ensure accuracy.

### Statistical analysis

Demographic characteristics of respondents were described using number and percentages. Student’s *t*-test and analyses of variance were conducted to test the statistical differences in professional identity, job satisfaction, burnout and turnover intention scores across different subgroups. Pearson’s correlation was used to assess the degree of correlation among different dimensions of these attributes. The above analyses were conducted using IBM SPSS Statistics 22.0 and the significance level was set at *P* < 0.05.

Structural equation modeling (SEM) performing on the AMOS 21.0 was conducted to further verify the hypothetical relationship among the four concepts of professional identity, job satisfaction, burnout, and turnover intention. This technique allowed us to decompose total effects of variables into direct and indirect effects [34]. As such, the results can provide a deep understanding for mechanisms and pathways of these variables. We also used a bias-corrected bootstrap 95% confidence interval (CI) to assess significance of total, direct and indirect effects [35].

Considering that sample characteristics, such as age, gender, education level, employment status, income level, and work hours, were associated with professional identity, job satisfaction, burnout and turnover intention, we adjusted these variables using classical multiple regression models (i.e., residual scores were obtained through regression) to control the influence of socio-demographic factors [36]. As a result, the regressed data was used in the SEM analysis. To assess the overall model fit, the four tests were used: RMSEA, goodness-of-fit index (GFI), comparative fit index (CFI), and Tucker-Lewis index (TLI). If GFI, CFI and TLI values above 0.90 and RMSEA values below 0.08, it indicates the model fit is acceptable [37].

## Results

### Characteristics of respondents

Of 3, 236 respondents, more than half of GPs were female. GPs aged from 31 to 40 accounted for 45.4% of the sample. Most respondents were married (85.6%) and had a bachelor degree (66.1%). Over 70% of GPs gave a low rating on their income level, and a small percentage of GPs (12.5%) had a senior profession title. About a quarter of respondents were employed as contracted workers and worked more than 8 h a day on average. Approximately half of GPs had worked for 4 to 10 years (Table 1).

### Differences in scores across different groups

Overall, the GPs gave a rating score of 51.22 ± 7.06, 37.24 ± 7.21, 60.12 ± 21.30, and 15.06 ± 4.33 for professional identity, job satisfaction, burnout, and turnover intention, respectively.

The differences in mean scores of professional identity, job satisfaction, burnout, and turnover intention among the different subgroups were presented in Table 1. Older GPs were more likely to give a positive rating on these four attributes (*P < 0.001*). Those who had a higher level of income also reported a higher professional identity, job satisfaction as well as lower burnout and turnover intention (*P < 0.001*).

Gender difference in scores was found in the job satisfaction, burnout and turnover intention (*P < 0.05*). There was significant difference in professional identity and burnout scores by marital status (*P < 0.001*). Respondents with different educational levels and working hours reported different scores in job satisfaction, burnout and turnover intention (*P < 0.001*). Those GPs with senior professional title tended to give a positive rating on professional identity, job satisfaction and burnout (*P < 0.01*). Difference in turnover intention was found among different employment type (*P < 0.001*). Those who had longer work tenure were more likely to reported a higher level of professional identity and lower level of burnout and turnover intention (*P < 0.01*).

### Correlations among professional identity, job satisfaction, burnout and turnover intention

Table 2 shows correlation coefficients between professional identity, job satisfaction, burnout and turnover intention. Overall, most of the coefficients were statistically significant at the *P < 0.001* level. Specifically, professional identity was positively associated with job satisfaction, as well as negatively associated with burnout and turnover intention. Job satisfaction had both negative associations with burnout and turnover intention. Additionally, the association between burnout and turnover intention was significantly positive.

**Table 2:** Correlation coefficients of professional identity, job satisfaction, burnout, and turnover intention

	1	2	3	4	5	6	7	8	9	10	11
**Professional identity**											
1. Professional value	1										
2. Professional efficacy	0.600^**^	1									
**Job satisfaction**											
3. Basic demand	0.203^**^	0.204^**^	1								
4. Relationship	0.344^**^	0.282^**^	0.675^**^	1							
5. Personal development	0.196^**^	0.198^**^	0.639^**^	0.669^**^	1						
**Burnout**											
6. Personal accomplishment	0.364^**^	0.207^**^	0.092^**^	0.200^**^	0.109^**^	1					
7. Depersonalization	−0.286^**^	−0.162^**^	−0.219^**^	−0.282^**^	−0.194^**^	−0.235^**^	1				
8. Emotional exhaustion	−0.171^**^	−0.054^**^	−0.340^**^	−0.376^**^	−0.283^**^	− 0.071^**^	0.536^**^	1			
**Turnover intention**											
9. TIS-I	−0.179^**^	− 0.131^**^	− 0.448^**^	− 0.411^**^	−0.407^**^	− 0.132^**^	0.313^**^	0.409^**^	1		
10. TIS-II	−0.061^**^	− 0.005	− 0.228^**^	− 0.175^**^	−0.217^**^	− 0.152^**^	0.051^**^	0.141^**^	0.466^**^	1	
11. TIS-III	−0.172^**^	−0.124^**^	− 0.363^**^	−0.344^**^	− 0.326^**^	−0.114^**^	0.308^**^	0.374^**^	0.764^**^	0.429^**^	1

Note: **: *p* < 0.001

*TIS-I* Turnover intention scale-I; *TIS-II* Turnover intention scale- II; *TIS-III* Turnover intention scale- III

### Synthesized relationship among professional identity, job satisfaction, burnout, and turnover intention: results from SEM

**Fig. 2:**
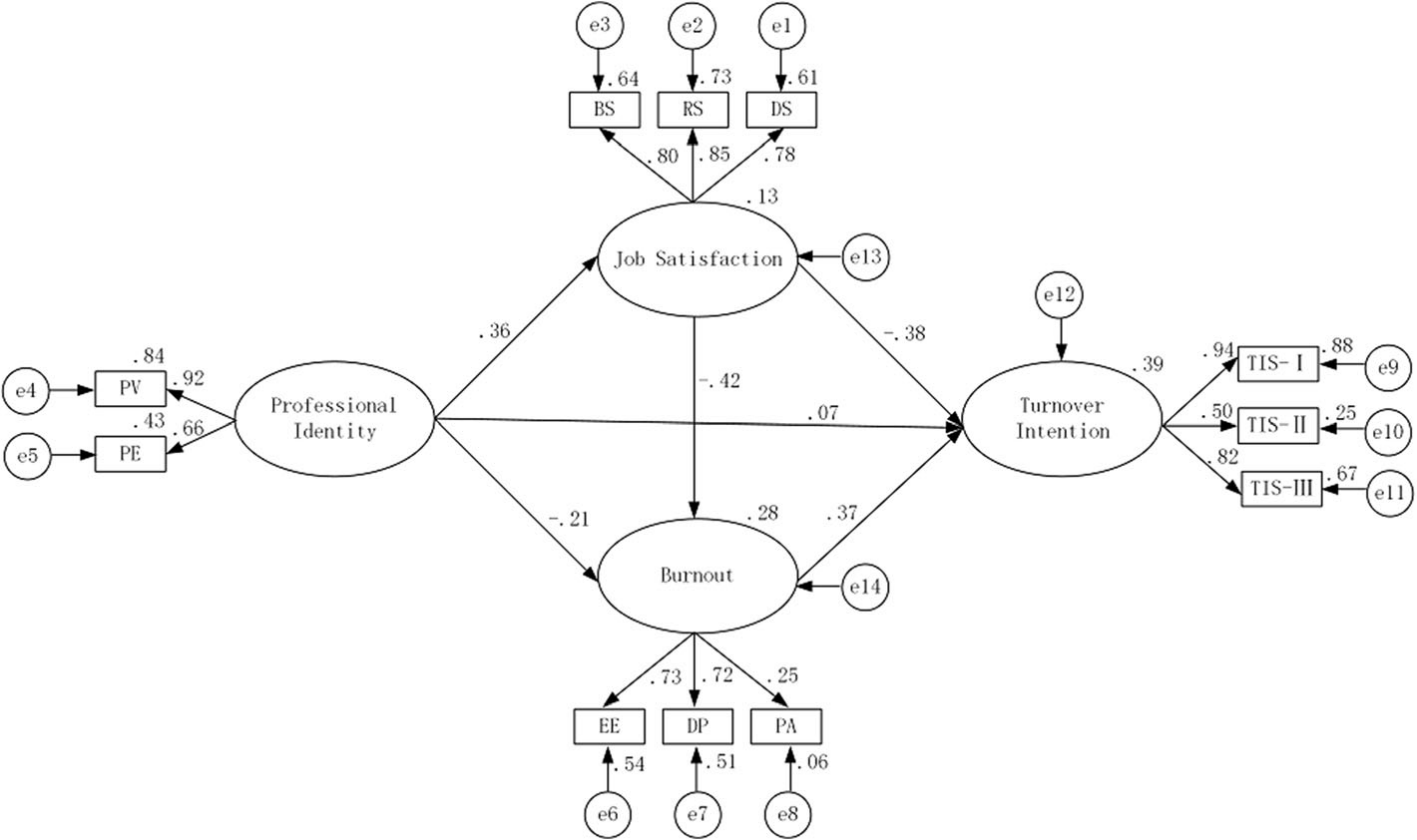
Model paths and standardized coefficients on synthesized relationship among professional identity, job satisfaction, burnout and turnover intention (PV: professional value, PE: professional efficacy, BS: basic satisfaction, RS: relationship satisfaction, DS: development satisfaction)

Figure 2 shows the model paths with standardized coefficients and Table 3 provides total, direct, and indirect effects of model paths. The significance of all the effects were examined using a 95% bootstrapped confidence interval estimate. The overall fit of the model was high based on the four goodness-of-fit indices: RMSEA = 0.008, CFI = 0.918, GFI = 0.934, TLI = 0.926.

**Table 3:** Total, direct, indirect effects of model paths

Model paths	Total effect	95% CI	Direct effects	95%CI	Indirect effects	95%CI
Professional identity→Job satisfaction	0.36^**^	(0.29, 0.42)	0.36^**^	(0.29, 0.42)	–	–
Professional identity→Burnout	−0.36^**^	(− 0.42, − 0.29)	−0.21^**^	(− 0.28, − 0.12)	−0.15^**^	(− 0.18, − 0.12)
Professional identity→Turnover intention	−0.20^*^	(− 0.26, − 0.17)	0.07	(−0.02, 0.11)	− 0.27^**^	(− 0.32, − 0.23)
Job satisfaction→Burnout	−0.42^**^	(− 0.47, − 0.36)	−0.42^**^	(− 0.47, − 0.36)	–	–
Job satisfaction→Turnover intention	−0.54^**^	(− 0.57, − 0.49)	−0.38^**^	(− 0.43, − 0.32)	−0.15^**^	(− 0.19, − 0.12)
Burnout→Turnover intention	0.37^**^	(0.32, 0.42)	0.37^**^	(0.32, 0.42)	–	–

*p < 0.05; ** *p* < 0.001

Overall, almost all effects of model paths were significant. Specifically, job satisfaction (*β* = − 0.42, *95%CI:* − 0.47 to − 0.36) and burnout (*β* = 0.37, *95%CI:* 0.32 to 0.42) had significant direct effects on turnover intention, but the direct effect of professional identity (*P* > 0.05) was not significant. As for indirect effects, it was found that the effect of professional identity (*β* = − 0.27, *95%CI*: − 0.32 to − 0.23) on turnover intention was mediated by both job satisfaction and burnout. Simultaneously, burnout was also acted as a mediator for the relationship between job satisfaction and turnover intention (*β* = − 0.15, *95%CI:* − 0.19 to − 0.12). In addition, we also found the mediation effect of job satisfaction on the relationship between professional identity and burnout (*β* = − 0.15, *95%CI:* − 0.18 to − 0.12).

## Discussion

The demand for primary healthcare services among the Chinese population is increasing and GPs constitute an essential part of the human resources need to provide these services. Training and retention of GPs are prudent strategies to improve quality care outcomes. Improvement in professional identity and job satisfaction, and decreasing in burnout and turnover intention among GPs are important for the provision of high-quality patient care. Identifying the interrelationships between professional identity, job satisfaction, burnout, and turnover intention among Chinese GPs would help the Chinese government to implement effective plans to strengthen the GPs workforce and improve the occupational attractiveness of GPs.

### Summary of main findings

This study has explored the status of turnover intention and the associations between professional identity, burnout and job satisfaction and turnover intention among urban GPs in China.

Overall, the study showed that 71.1% of Chinese urban GPs reported moderate or higher levels of intention to quit their jobs, which is higher than that of village doctors (36.8%) [38] and health inspectors in China (45.3%) [22]. The turnover intention rate in this study is also higher than the reporting rates found in developed countries (e.g., New Zealand, Sweden, and Finland) [39–41]. The differences might be at least partly attributable to the participants’ characteristics (i.e., age, education, income level, practice setting (rural/urban areas), sample size, and the measurement of turnover intention). In addition, the study indicated that 37.5% Chinese urban GPs were dissatisfied with their current job, which is higher than a study performed in Denmark (22.1%) [42]. More than half of GPs (51.6%) reported higher levels of job satisfaction. The rate of high job satisfaction in the current study is in the middle of reporting rates found in developed countries (e.g., Germany (42%) [43] and Canada (72%)) [44].

### Job satisfaction had direct and indirect effects on turnover intention

Job satisfaction was an important factor that had both direct and indirect effects on turnover intention. Job satisfaction was associated with an individual’s perceptions and evaluation of the job, and this perception is influenced by person-specific status such as demands, values and expectations. Therefore, GPs with higher levels of job satisfaction tended to have higher enthusiasm for work and achieve a greater accomplishment from works they do, which in turn reduced the incidence of burnout. Moreover, previous literature found that burnout increased the risk of intention to leave [45]; thus, in this study the effect of job satisfaction on turnover intention can be mediated by burnout, which supported our hypotheses.

### Burnout had a directly positive effect on turnover intention

In our study, we found that burnout had a directly positive effect on turnover intention. Similar to previous studies [46, 47], this study showed that burnout positively linked with turnover intention. According to the 2014 Chinese Medical Doctors Association report, 66% of GPs worked more than 40 h per week [48]. In China, the income of GPs was less than average income and far less than specialists’ income, especially among those working in primary heath institutions. Chinese GPs were paid within a reference range set by the government though the range may vary depending on the area and their work tenure [11]. Due to the heavy workload, and low level of income, GPs often report poor experience on work, and they sometimes have faced distrust from patients in the Chinese primary care sector, which further resulted in their burnout [49–51]. Based on the previously published literatures [52, 53], individuals who was in a status of burnout in a long time was unable to cope with the work pressure, and eventually led to resignation. This finding suggests that a reduction in burnout could serve as an effective way to reduce turnover intention, and provides new insights for future studies on ways to reduce the occupational burnout and indirectly address high turnover intention problems among GPs. Actions such as reducing the work intensity, increasing the wages of GPs, providing career development opportunities for GPs, and improving the skill and health service delivery ability of GPs are warranted to reduce the level of burnout of GPs.

### The mediating effects of job satisfaction and burnout on GPs’ turnover intention

Our results were in agreement with previous studies [22, 54, 55] showing that professional identity had a positive effect on job satisfaction and a negative effect on burnout. When individuals had a high degree of identity with their career, they would devote more time and efforts and vigour to their practice field, and the job dissatisfaction resulted from work environment and condition may be reduced or even eliminated [56]. Moreover, individuals had a high professional self-concept (a perception of oneself as a member of the profession), which could reduce the incidence of burnout [57]. Combining the significant associations of turnover intention with job satisfaction and burnout, it is not difficult to understand indirect effect of professional identity on turnover intention.

Intriguingly, we found that professional identity had no direct effect on turnover intention, which was inconsistent with previous studies [54, 58]. One possible interpretation could be that in this study, professional identity focused on the GPs’ perception on value of healthcare work itself, instead of their current position. In current context of Chinese primary care institutions, GPs often undertook a large amount of basic medical and public health service delivery, but usually earned fewer wage compared with doctors in hospitals. Therefore, those GPs with a high level of professional identity may also plan to leave their current job and enter advanced health institutions.

### Strengths and limitations

This study has several strengths. First, to date, this is the first study to assess the mediating effects of job satisfaction and burnout on turnover intention among Chinese urban GPs based on a national representative sample. Second, we examined the interrelationships between professional identity, job satisfaction, burnout, and turnover intention. The results of our study not only can provide a new insight for GPs’ intention to leave their present jobs, but also serve as new methods to address the high turnover issues among GPs in China. Future studies on constructing different models to examine the mechanism of professional identity on turnover intention by introducing other mediator factors are needed.

This study had several limitations. First, the analysis of cross-sectional data limited our ability to establish causal relationships among study variables. In addition, variables in our study were collected based on self-reported measurement, which was a potential source of measurement error. However, previous studies have suggested that there was qualified agreement between online self-reported and objected measured data, which were sufficiently valid for epidemiological investigations [59].

### Implications for research and practice

GPs play important roles in enhancing quality of primary health care in the health care system. Awareness and management of turnover intention among GPs may stabilize the workforce in the primary health care system. Thus, policy makers of the country’s health care service should take actions to improve job satisfaction and reduce the burnout of GPs. In addition, measures should be taken to improve GPs’ professional identity that could help to increase job satisfaction and reduce occupational burnout.

## Conclusions

In conclusion, three affecting paths of turnover intention were identified in our study. Job satisfaction, professional identity and burnout were all strong predicators of turnover intention. Professional identity had an indirect effect on turnover intention through job satisfaction and burnout instead of affecting turnover intention directly. Job satisfaction and burnout had significantly direct effects on turnover intention.

## Supplementary Information

**Additional file 1.** Questionnaire.

## Data Availability

Data may be made available by contacting the corresponding author.

## Abbreviations

CHC: Community health center
CHI: Community health institution
CFI: Comparative fit index
CHS: Community health service
GFI: Goodness-of-fit index
GP: General practitioner
IFI: Incremental fit index
MBI-HSS: Maslach burnout inventory-human services survey
NFI: Normed fit index
RMSEA: Root mean square error of approximation
SEM: Structural equation modeling
THC: Township health center
TLI: Tucker-Lewis index
